# Identification, quantification, and antidepressant‐like evaluation of anthocyanin‐rich extracts from different dietary berries

**DOI:** 10.1002/fsn3.4280

**Published:** 2024-06-14

**Authors:** Jun Wang, Jie Cheng, Ji‐Xiao Zhu, Guang‐Hui Xu, Wei‐Feng Huang, Li‐Tao Yi

**Affiliations:** ^1^ Department of Chemical and Pharmaceutical Engineering, College of Chemical Engineering Huaqiao University Xiamen People's Republic of China; ^2^ Research Center of Traditional Chinese Medicine Resources and Ethnic Medicine Jiangxi University of Chinese Medicine Nanchang People's Republic of China; ^3^ Xiamen Medicine Research Institute Xiamen People's Republic of China; ^4^ Department of Gastroenterology and Hepatology The First Affiliated Hospital of Xiamen University, School of Medicine, Xiamen University Xiamen People's Republic of China; ^5^ Institute of Pharmaceutical Engineering, Huaqiao University Xiamen People's Republic of China; ^6^ Fujian Provincial Key Laboratory of Biochemical Technology Huaqiao University Xiamen People's Republic of China

**Keywords:** anthocyanin, antidepressant, black mulberry, blueberry

## Abstract

Berries are highly valued for their rich phenolic content, particularly anthocyanins, which are known for their antioxidative properties and potential effectiveness against depression. This study identified and quantified anthocyanin profiles in blueberry, blackberry, black mulberry, and cranberry using ultra‐performance liquid chromatography–mass spectrometry (UPLC–MS), followed by in vivo evaluation of their antidepressant‐like activities. Blueberry and black mulberry showed significant levels of cyanidin‐3‐galactoside and cyanidin. Acute supplementation with these berries decreased serum corticosterone levels and increased monoamine neurotransmitters, while chronic supplementation enhanced antioxidative activity and reduced neuroinflammation in the hippocampus of mice. These findings indicate that the neurochemical, antioxidative, and anti‐inflammatory effects of blueberry and black mulberry mediate their therapeutic role against depression. Berries rich in cyanidin‐3‐galactoside and cyanidin may be valuable in developing functional foods, dietary supplements, and pharmacological formulations for mental health improvement.

## Graphical abstract

This study identified and quantified anthocyanin profiles in blueberry, blackberry, black mulberry, and cranberry using ultra‐performance liquid chromatography–mass spectrometry (UPLC–MS). An animal study showed that blueberry and black mulberry possessed the antidepressant‐like effects in acute and chronic supplement procedure. Biochemical assays showed that the neurochemical, antioxidative, and anti‐inflammatory effects of blueberry and black mulberry mediated their therapeutic role against depression.

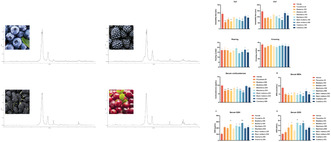

## 1 INTRODUCTION

Berries, widely recognized as highly nutritious fruits, are consumed around the world for their delightful flavors and impressive health benefits. They are small, soft, round fruits that typically come in various shades of red, blue, and purple. Common varieties include strawberries, blueberries, raspberries, blackberries, and cranberries, among others. The popularity of berries can be attributed to their versatility and ease of incorporation into a variety of dietary patterns, ranging from fresh consumption to their use in processed forms like jams, juices, smoothies, and desserts.

Anthocyanins are pigments that give berries their vibrant red, purple, and blue hues. They are typically found in the skin and flesh of these fruits and vary in type and concentration depending on the specific berry species, environmental conditions, and cultivation practices (Ponder et al., 2021). Scientifically, anthocyanins are appreciated not only for their colorant properties but also for their array of health‐promoting effects. Structurally, anthocyanins are glycosides of anthocyanidins, where the sugar part is typically linked to the oxygen atom of the C‐ring of the anthocyanidin backbone. This structural variety contributes to their diverse range of colors and stability, which can be influenced by pH, light, temperature, and the presence of other compounds (Khoo et al., 2017).

Research has extensively documented the antioxidant properties of anthocyanins (Ali et al., 2022; Gowd et al., 2019; Li et al., 2024). These molecules can donate electrons to neutralize free radicals, reducing oxidative stress and thereby attenuating the molecular damage associated with chronic diseases, such as cancer, cardiovascular diseases, and age‐related neurological declines (Saini et al., 2024; Zaa et al., 2023). Furthermore, anthocyanins have been shown to modulate a number of cellular pathways to exert anti‐inflammatory effects (Brunelle et al., 2024), enhancing their appeal as dietary components for health maintenance and disease prevention.

Oxidative stress and inflammation are crucially involved in the pathophysiology of depression. Oxidative stress refers to the imbalance between free radicals and antioxidants in the body, leading to cellular damage (Bakunina et al., 2015; Correia et al., 2023). This process can disrupt neuronal function and plasticity, contributing to the onset and progression of depression (Chen, Lu, et al., 2024). Concurrently, chronic inflammation has been identified as a key factor in depressive disorders (Zagaria et al., 2024). Pro‐inflammatory cytokines can influence neurotransmitter metabolism, neuroendocrine function, and neural plasticity, thereby exacerbating depressive symptoms. The interplay between oxidative stress and inflammation creates a detrimental cycle that can further deteriorate mental health. Neuroprotective effects are another prominent aspect of anthocyanins (Chen, Xie, et al., 2024). Dietary intake of these compounds has been associated with enhanced cognitive function and a reduced risk of neurodegenerative diseases (Zaa et al., 2023). Studies suggest that anthocyanins can cross the blood–brain barrier (Hribar & Ulrih, 2014), where they exert antioxidant and anti‐inflammatory actions within the brain. They are thought to protect neurons by inhibiting neuroinflammation, modulating neuronal signaling, and improving blood flow to the brain, which could be particularly beneficial in aging populations (Khan et al., 2019; Zhang & Jing, 2023).

Berries possess potential antioxidative, anti‐inflammatory, and neuroprotective roles due to their rich and diverse anthocyanins. However, few studies have compared the antidepressant‐like effects of different types of berries in vivo. Therefore, in the present study, we tried to identify and quantify the representative anthocyanin from four types of berries including blueberry, blackberry, black mulberry, and cranberry. Then, the potent antidepressant‐like effects were evaluated from the four berries followed by their neurochemical and antioxidative regulatory activities. Furthermore, we used chronic supplement procedure to verify the antidepressant‐like effects and the neuroinflammatory activity. Through these investigations, the study seeks to substantiate the therapeutic potential of berries against neurodegenerative and neuropsychiatric disorders, thereby supporting their use in dietary strategies and nutraceutical applications aimed at enhancing cognitive and mental health.

## 2 MATERIALS AND METHODS

### 2.1 Extraction of anthocyanin from several berry fruits

Berries including blueberry (*Vaccinium* spp., Yunnan), blackberry (*Rubus fruticosus*, Yunnan), black mulberry (*Morus alba L*., Xinjiang), and cranberry (*Vaccinium macrocarpon*, Heilongjiang) were purchased from local supermarkets within China. The moisture content of each berry type was quantified using the standard oven‐drying method to establish baseline hydration levels. For the extraction of anthocyanins, an optimized ultrasound‐assisted extraction methodology was employed, which was refined through preliminary trials to enhance yield and purity. Initial preparation involved homogenizing the berries using a mechanical juicer to break down cellular structures and facilitate solvent access. The homogenates were then incubated with a solvent system comprising 60% ethanol and 0.1 M hydrochloric acid, mixed in a volume‐to‐volume ratio of 6:1. This mixture was subjected to ultrasound‐assisted extraction for 60 min using an ultrasonic bath set at a power of 140 watts and a temperature maintained at 30°C to optimize the extraction efficiency without degrading sensitive anthocyanin structures. Following extraction, the mixtures were filtered to remove particulate matter. The filtrates were then concentrated under reduced pressure to remove the majority of the solvent, resulting in a extract. This extract, referred to as anthocyanin‐rich concentrate, contains the bioactive compounds of interest from each type of berry.

### 2.2 High‐performance liquid chromatography (HPLC) quantification of anthocyanins

Quantitative analysis of anthocyanins in extracts was performed using 15 selected standards: Cyanidin‐3,5‐diglucoside, Cyanidin‐3‐galactoside, Delphinidin, Procyanidin B4, Cyanidin, Procyanidin B2, Petunidin, Pelargonidin, Peonidin, Malvidin, Rutin, Luteolin, Quercetin, Isorhamnetin, and Kaempferol (Table 1). These analytes were chosen based on their prevalence in various botanical sources and their significance in nutritional studies. All standards were procured from Sigma‐Aldrich (St. Louis, USA). To construct calibration curves, mixed standard solutions were prepared in concentrations ranging from 50 to 2000 ng/mL. Sample preparation involved accurately weighing 100 mg of the dried plant material, followed by dual extractions using 1 mL of a solvent mixture composed of methanol, water, and formic acid (70:30:1, v/v/v). This mixture was selected to optimize the solubilization of anthocyanins. Each sample underwent vigorous vortexing and ultrasonic treatment for 20 min to enhance extraction efficiency. Subsequently, the mixtures were centrifuged at 12,000 rpm (revolutions per minute) for 10 min, and the clear supernatants were filtered through a 0.22‐μm nylon membrane filter. Solid‐phase extraction (SPE) was conducted using hydrophilic–lipophilic balance (HLB‐SPE) columns to purify the extracts. The SPE procedure involved preconditioning the column with 1 mL of methanol followed by 1 mL of deionized water. The anthocyanin‐rich supernatant was then percolated through the column, followed by washing with 1 mL of water. Elution of anthocyanins was achieved with 1 mL of methanol containing 5% formic acid. The eluate was collected, dried under a stream of nitrogen, freeze‐dried, and reconstituted in 0.2 mL of methanol for further analysis. Chromatographic separation was achieved on a Waters ACQUITY UPLC BEH C18 column (1.8 μm, 2.1 × 50 mm) maintained at 40°C. The mobile phase comprised water and acetonitrile, each containing 0.1% formic acid. The flow rate was set at 0.3 mL/min with an injection volume of 2 μL. The gradient commenced with 95% water and shifted to 70% at 6.0 min, reaching 5% at 7.0 min, and held until 8.1 min, before returning to initial conditions at 10.0 min. Analysis was conducted using an ultra‐performance liquid chromatography (UPLC) coupled to an Orbitrap mass spectrometer (Vanquish UPLC and Q Exactive (QE)/MS), optimizing detection and quantification of the targeted analytes.

**TABLE 1:** The calibration curve of the 15 standards by UPLC.

Standard name	CAS	Molecular formula	Calibration	*R* ^2^
Malvidin	643‐84‐5	C17H15ClO7	Y = 2.306e4X	0.9995
Pelargonidin	134‐04‐3	C15H11ClO5	Y = 6.456e4X	0.9978
Delphinidin	528‐53‐0	C15H11ClO7	Y = 8.929e3X	0.9996
Peonidin	134‐01‐0	C16H13ClO6	Y = 4.378e4X	0.9998
Cyanidin‐3‐galactoside	27661‐36‐5	C21H21ClO11	Y = 6.731e4X	0.9995
Isorhamnetin	480‐19‐3	C16H12O7	Y = 7.898e4X	0.9993
Cyanidin	528‐58‐5	C15H11ClO6	Y = 4.053e4X	1
Cyanidin‐3,5‐diglucoside	2611‐67‐8	C27H31ClO16	Y = 2.041e4X	1
Petunidin	1429‐30‐7	C16H13ClO7	Y = 2.314e4X	0.9998
Quercetin	117‐39‐5	C15H10O7	Y = 6.944e4X	0.9977
Rutin	153‐18‐4	C27H30O16	Y = 2.316e4X	0.9998
Procyanidin B4	29106‐51‐2	C30H26O12	Y = 6.552e3X	0.9998
Luteolin	491‐70‐3	C15H10O6	Y = 9.19e4X	0.9996
Procyanidin B2	29106‐49‐8	C30H26O12	Y = 1.574e4X	1
Kaempferol	520‐18‐3	C15H10O6	Y = 6.011e4X	0.9998

### 2.3 Animals

Male Institute of Cancer Research (ICR) mice, each weighing between 24 and 26 g and of specific pathogen‐free (SPF) status, were procured from Shanghai Slack Laboratories. These mice were subsequently housed in the Experimental Animal Center under controlled conditions, maintaining a temperature of 23–27°C and a relative humidity (RH) of 40%–60%. A 12‐h light–dark cycle was established. The mice were given ad libitum access to water and standard laboratory chow and underwent a 1‐week acclimatization period before initiation of the experiments. All experimental procedures received prior approval from the Huaqiao University Institutional Animal Care and Use Committee, Approval No. A2024002, and were conducted in strict accordance with guidelines set by the China Council on Animal Care.

### 2.4 Reagents

Bicinchoninic acid (BCA, PC0020), malondialdehyde (MDA, BC0025), glutathione (GSH, BC1175), and superoxide dismutase (SOD, BC5165) kits were purchased from Solarbio Life Sciences (Beijing, China). Mouse interleukin‐1 beta (IL‐1β) ELISA kit (BP80277), mouse interleukin‐6 (IL‐6) ELISA kit (BP80293), and mouse tumor necrosis factor‐alpha (TNF‐α) ELISA kit (BP80317) were purchased from Purdue Bioscience (New York, USA). Mouse corticosterone ELISA kit (501320) was purchased from Cayman Chemical (Ann Arbor, USA). Serotonin ELISA kit (ADI‐900‐175) and Dopamine ELISA kit (ENZ‐KIT188) were purchased from Enzo Life Sciences (Farmingdale, USA). Norepinephrine ELISA kit (KA3836) was purchased from Novus Biologicals (Centennial, USA).

### 2.5 Acute supplement experiment

Animals were divided into seven experimental groups: a control group receiving 0.9% saline, a fluoxetine group dosed at 20 mg/kg, blueberry supplement groups (250, 500 mg/kg), blackberry supplement groups (250, 500 mg/kg), black mulberry supplement groups (250, 500 mg/kg), and cranberry supplement groups (250, 500 mg/kg), respectively. The dosing volume was standardized at 10 mL/kg body weight. To assess the potential antidepressant‐like effects of the extracts mentioned above, it was administered orally 60 min prior to conducting the open‐field test (OFT), tail suspension test (TST), and forced swimming test (FST).

### 2.6 Open‐field test

Locomotor activity was evaluated using the open‐field test. Mice received the same dosages that demonstrated anti‐immobility effects in the tail suspension test. The test was conducted in a wooden box, dimensions 40 × 40 × 30 cm, with its floor marked into 25 equal squares (each 8 × 8 cm). During a 3‐min session, both the number of squares each mouse crossed with all paws (crossings) and the instances of standing on hind legs (rearings) were recorded. Between tests, the apparatus was thoroughly cleaned with detergent and allowed to dry to prevent olfactory cues. All test sessions were video recorded and later analyzed by an observer who was blinded to the supplement groups.

### 2.7 Tail suspension test

The tail suspension test was conducted with modifications to the method originally described by Steru et al. (1985). Mice were individually suspended by the tail using a clamp placed 1 cm from the tip, within an enclosed box measuring 25 × 25 × 30 cm. The heads of the mice were maintained 5 cm above the box floor. Testing occurred in a dimly lit room to minimize external stimuli. Each mouse was suspended for a total of 6 min, with the duration of immobility recorded during the final 4‐min interval. Immobility was defined as the absence of movement, with the mice hanging passively and motionless. All test sessions were video recorded and subsequently scored by an observer who was blinded to the supplement groups.

### 2.8 Forced swimming test

The forced swimming test was performed with slight modifications to the protocols described in previous studies (Bourin et al., 2004; Porsolt et al., 1977). Mice were individually placed in a glass cylinder measuring 20 cm in height and 14 cm in diameter, filled with water to a height of 10 cm at a temperature of 25 ± 2°C. Each animal was required to swim for a total of 6 min, during which the duration of immobility was recorded in the last 4‐min interval. Immobility was defined as the mouse floating in the water without struggling, performing only minimal movements necessary to maintain its head above water. The sessions were video recorded and subsequently analyzed by an observer who was blinded to the supplement.

### 2.9 Serum corticosterone assay

Following the forced swimming test, blood samples were collected from the mice. Serum was obtained by centrifuging these samples at 3000 × g for 10 min. The serum was then used to measure corticosterone levels using an ELISA kit according to the manufacturer's protocol (Enzo Life Sciences, Farmingdale, NY, USA).

### 2.10 Biochemical and enzyme‐linked immunosorbent assay (ELISA) measurement

The quantification of monoamine neurotransmitters in the hippocampal tissue was conducted using commercial ELISA kits. All the procedures were homogenized according to the instructions of the kits.

### 2.11 Chronic mild stress (CMS)

The CMS protocol was administered according to methods previously described. In brief, the stress regimen, implemented weekly, included: food and water deprivation, placement of an empty bottle, transfer to a soiled cage, rapid alterations between light and dark every 2 h, space confinement, a 45° cage tilt, continuous overnight illumination, and exposure to predator sounds. Each stressor was applied independently and continuously, both day and night. Control animals were housed in a separate facility to avoid any interaction with the stressed groups. To prevent habituation and maintain the unpredictability of stress exposures, all stressors were randomly assigned over each week and this schedule was consistently applied over the duration of the eight‐week study. Following 4 weeks of exposure to CMS, mice from both stressed and control groups were subdivided into matched subgroups (*n* = 8 each) for supplements and another 4 weeks of CMS, based on their sucrose preference test results.

### 2.12 Chronic supplement experiment

The mice were randomly divided into four groups: the Control–vehicle group, CMS–vehicle group, CMS–blueberry group, and CMS–black mulberry group. Blueberry and black mulberry were orally administered at 500 mg/kg once a day for 28 days. All these extracts were administered in a volume of 10 mL/kg.

### 2.13 Sucrose preference test

First of all, a sucrose adaptation procedure began 3 days prior to the start of the experiment. During the formal sucrose preference test, in the first 24 h, the mice had access to two bottles containing 1% sucrose solution. This was followed by a 24‐h period where the mice were given one bottle of 1% sucrose solution and one bottle of water, with the positions of these bottles switched every 12 h to prevent location preference. In the measuring phase of the experiment, each cage was equipped with one bottle of 1% sucrose solution and one bottle of water, with their positions alternated every 12 h. The consumption of both the sucrose solution and water was monitored over a 24‐h period to calculate the sucrose preference (%).

### 2.14 Reverse transcription‐polymerase chain reaction (RT‐PCR)

Hippocampal tissues were powered in liquid nitrogen and stored for further measurement. Total RNA was isolated from hippocampal tissue utilizing Trizol reagent. The synthesis of complementary DNA (cDNA) was performed employing reverse transcriptase. For the quantification of gene expression, quantitative PCR (qPCR) assays were conducted. Specific primers were used to amplify IL‐1β, IL‐6, TNF‐α, and glyceraldehyde‐3‐phosphate dehydrogenase (GAPDH) as an internal control for normalization. The sequences for these primers are as follows: IL‐1β (forward: 5′‐TGCCACCTTTTGACAGTGATG‐3′, reverse: 5′‐TGATGTGCTGCTGCGAGATT‐3′), IL‐6 (forward: 5′‐CCCCAATTTCCAATGCTCC‐3′, reverse: 5′‐CGCACTAGGTTTGCCGAGTA‐3′), TNF‐α (forward: 5′‐GATCGGTCCCCAAAGGGATG‐3′, reverse: 5′‐CCACTTGGTGGTTTGTGAGTG‐3′), and GAPDH (forward: 5′‐GGGTGTGAACCACGAGAAAT‐3′, reverse: 5′‐GGAAGAATGGGAGTTGCTGT‐3′). The detection of fluorescence signals occurred at the conclusion of each cycle of amplification. Relative quantification of gene expression was performed using the delta–delta threshold cycle (2^−ΔΔCT^) method, with normalization to GAPDH messenger RNA (mRNA) levels to account for variability in input RNA amounts and reverse transcription efficiency.

### 2.15 Biochemical and ELISA measurement

After blood collection, serum was obtained by centrifugation at 300 g, 4°C for 15 min. Hippocampal tissues were homogenized according to the instructions of the kits. All the detected procedures were also strictly followed according to the instructions of the kits.

### 2.16 Statistical analyses

All data are presented as mean ± standard error of the mean (SEM). Statistical analyses were conducted using one‐way analysis of variance (ANOVA) followed by Dunnett's post hoc test for multiple comparisons. A *p*‐value less than .05 was deemed statistically significant.

## 3 RESULTS

### 3.1 The contents of several representative anthocyanins in the extracts of different berries

**FIGURE 1:**
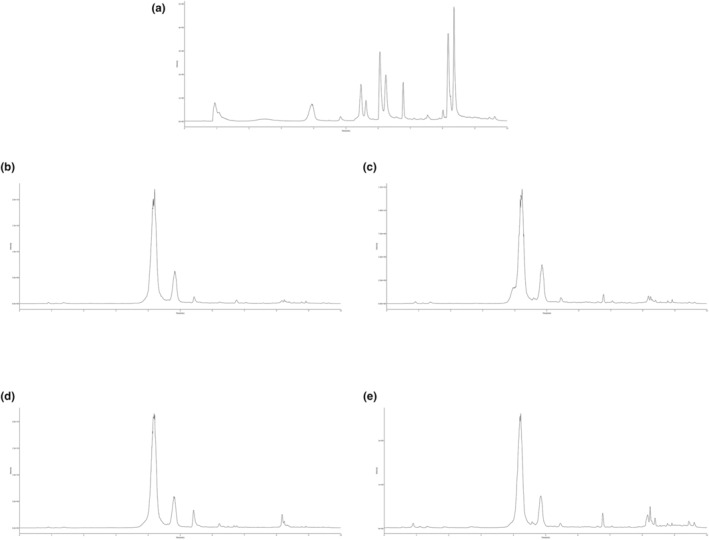
The total ion flow chromatogram of (a) mixed standards, (b) blueberry, (c) blackberry, (d) black mulberry, and (e) cranberry by UPLC–Q Exactive/MS.

To delineate the anthocyanin profiles within the rich extracts derived from various berries, UPLC–MS was employed. This analytical approach enabled the quantification of 15 key anthocyanins present in the extracts. The ion flow chromatograms, depicted in Figure 1, illustrate the chromatographic separation of standards alongside the extracts from four distinct berry types. As detailed in Table 2, a diverse array of anthocyanins was identified and quantified across the berry samples. Notably, blueberry and black mulberry extract exhibited significant concentrations of cyanidin‐3‐galactoside (30,310.28 and 30,003.25 μg/g, respectively) and cyanidin (1061.13 and 3303.94 μg/g, respectively). Similarly, blackberry displayed high levels of cyanidin‐3‐galactoside (17,598.43 μg/g) and rutin (615.65 μg/g). In contrast, cranberry showcased the highest level of cyanidin‐3‐galactoside among the sampled berries at 3382.95 μg/g. The ultrahigh‐performance liquid chromatography–mass spectrometry (UHPLC–MS) methodology facilitated a comprehensive analysis of anthocyanin compounds in the extracts from blueberry, blackberry, black mulberry, and cranberry. The identification and quantification of these bioactive compounds underscore their potential utility in the nutraceutical and pharmaceutical industries. Specifically, anthocyanin‐rich extracts may be leveraged in the development of functional foods, dietary supplements, and pharmacological formulations.

**TABLE 2:** The 15 different anthocyanins in the different berries by UPLC (μg/g).

Standards	Blueberry	Blackberry	Black mulberry	Cranberry
Cyanidin‐3,5‐diglucoside	0	0	0	0
Cyanidin‐3‐galactoside	30310.28	17598.43	30003.25	3382.95
Delphinidin	0.11	0.08	1.38	0.60
Procyanidin B4	0.07	269.48	0.37	60.43
Cyanidin	1061.13	432.52	3303.94	70.91
Procyanidin B2	0.19	71.52	69.13	21.54
Petunidin	0.53	0.68	10.79	0.38
Pelargonidin	1.74	1.12	9.16	0.32
Peonidin	122.64	46.24	451.55	7.16
Malvidin	0.22	0.62	35.18	0.83
Rutin	37.46	615.65	127.63	207.25
Luteolin	3.50	4.70	14.90	3.59
Quercetin	98.64	209.78	988.47	38.52
Isorhamnetin	14.51	20.86	28.19	7.56
Kaempferol	1.57	3.94	50.02	3.05

### 3.2 Effects of different berry extracts on stress‐induced depressive‐like behaviors

**FIGURE 2:**
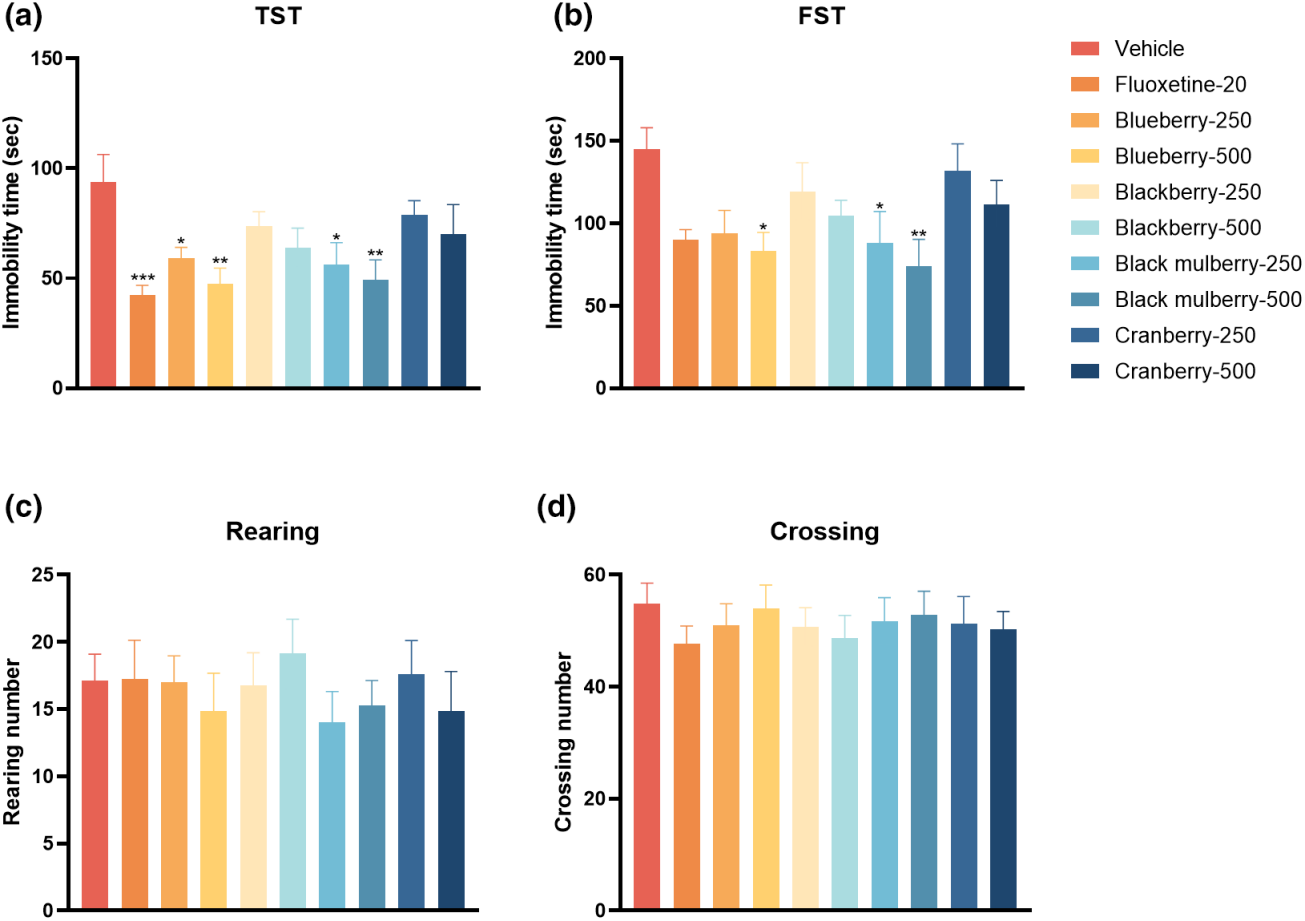
Effects of four different dietary berries after acute administration on behaviors in tail suspension test, forced swimming test, and open‐field test in mice (*n* = 8). (a) The immobility time in the tail suspension test. (b) The immobility time in the forced swimming test. (c) The rearing number in the open‐field test. (d) The crossing number in the open‐field test. Significance was assessed using a one‐way ANOVA followed by Dunnett's post hoc test. *p < .05, **p < .01, and ***p < .001 vs vehicle group.

To determine whether different berry extracts possess the antidepressant‐like effects, the stressed models TST and FST were performed in mice, as TST and FST tests are typically used to screen antidepressants after acute supplement procedure. According to Figure 2a, blueberry and black mulberry both at 250 and 500 mg/kg decreased the immobility time in TST. In addition, blueberry at 500 mg/kg as well as black mulberry at 250 and 500 mg/kg decreased the immobility time in FST (Figure 2b). On the contrary, blackberry and cranberry did not exert any alteration in immobility time in both the TST and FST. Furthermore, to rule out the positive effects in TST and FST, locomotor activity in OFT was evaluated (Figure 2c,d). The results showed that neither blueberry nor black mulberry changed the rearing or crossing number in OFT, suggesting that the reduction of immobility time in TST and FST was not due to the excitability of central nervous system (CNS). Taken together, these data indicated that blueberry and black mulberry possessed the antidepressant‐like effects in mice.

### 3.3 Effects of different berry extracts on serum corticosterone, MDA, GSH, and SOD levels

**FIGURE 3:**
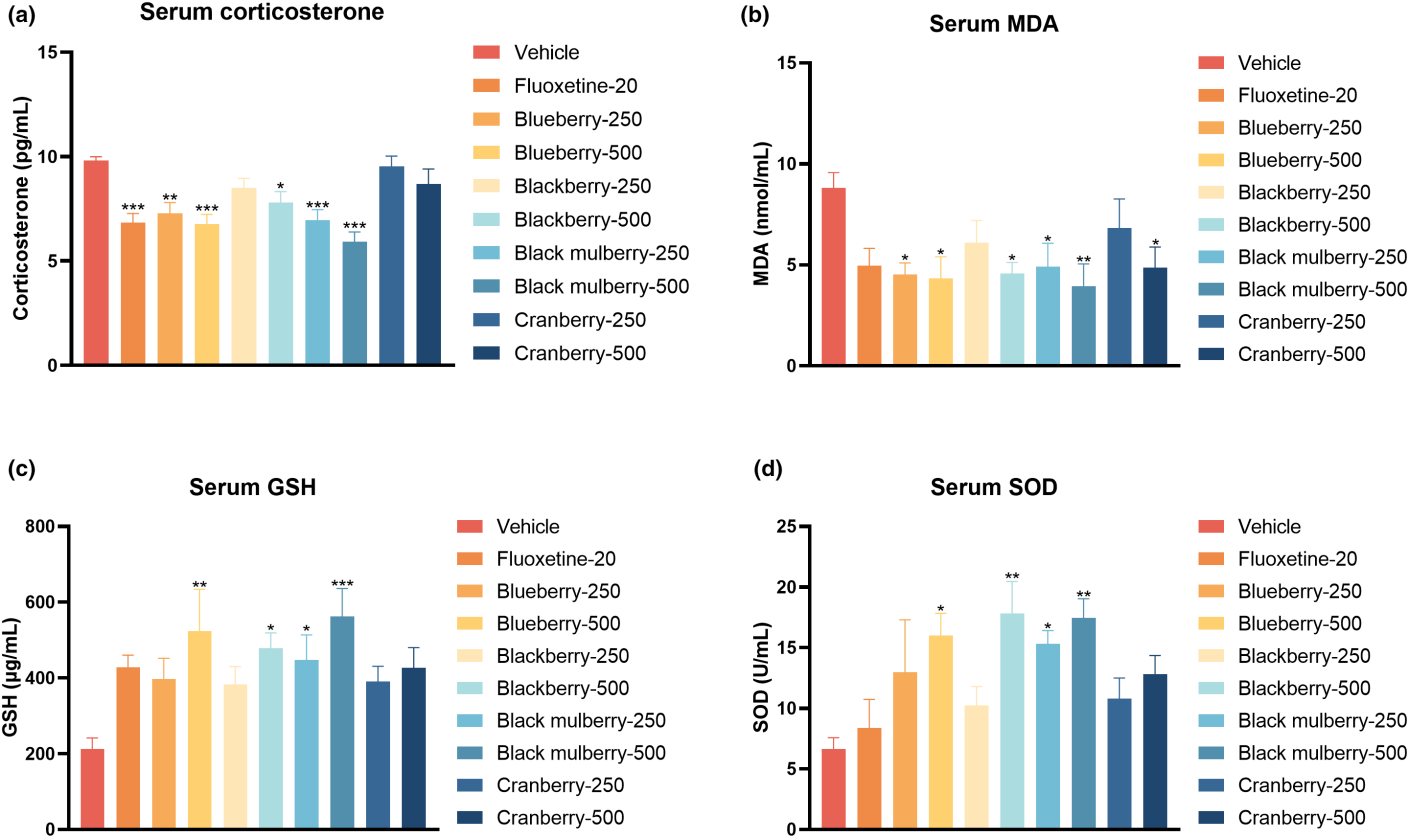
Effects of four different dietary berries after acute administration on serum (a) corticosterone, (b) MDA, (c) GSH, and (d) SOD levels (*n* = 8). Significance was assessed using a one‐way ANOVA followed by Dunnett's post hoc test. *p < .05, **p < .01, and *** *p* < .001 vs vehicle group.

Subsequently, we used the ELISA method to detect serum corticosterone levels in mice administered with different berry extracts. As shown in Figure 3a, blueberry and black mulberry both at 250 and 500 mg/kg decreased the serum corticosterone concentrations after acute stress exposure. In addition, blackberry at 500 mg/kg and positive drug fluoxetine at 20 mg/kg also decreased the corticosterone concentrations in serum. Then, oxidative stress indicators, such as MDA, GSH, and SOD, were evaluated in the serum by biochemical kits. As shown in Figure 3b, blueberry and black mulberry both at 250 and 500 mg/kg as well as blackberry and cranberry at 500 mg/kg decreased the MDA content in serum. In contrast, black mulberry at 250 and 500 mg/kg, blackberry at 500 mg/kg, and cranberry at 500 mg/kg increased GSH content and SOD activity in serum (Figure 3c,d).

### 3.4 Effects of different berry extracts on hippocampal monoamine neurotransmitter levels

The monoamine neurotransmitter contents in the hippocampus were assessed by ELISA. First, as shown in Table 3, only black mulberry at 500 mg/kg increased the 5‐hydroxytryptamine (5‐HT) content in the hippocampus of mice compared with vehicle group. Similarly, fluoxetine also increased 5‐HT contents in the hippocampus. In addition, 500 mg/kg blueberry, black mulberry, and cranberry increased the norepinephrine (NE) content in the hippocampus of mice compared with vehicle group. There was a trend toward increased NE content in blueberry and blackberry at the 250 mg/kg group, but it was not statistically significant. Lastly, there was no significant alteration in dopamine (DA) content after all the four berry extracts.

**TABLE 3:** The concentrations of monoamine neurotransmitters in the hippocampus.

Group	5‐HT (ng/g tissue)	NE (ng/g tissue)	DA (ng/g tissue)
Vehicle	603.5 ± 227.9	167.7 ± 40.9	45.8 ± 21.4
Fluoxetine‐20	1011.5 ± 346.9*	242.4 ± 188.8	56.0 ± 18.0
Blueberry‐250	937.9 ± 303.9	331.9 ± 185.8	63.1 ± 31.0
Blueberry‐500	976.0 ± 328.4	363.3 ± 130.3*	56.5 ± 26.2
Blackberry‐250	855.0 ± 262.5	334.8 ± 92.7	66.5 ± 34.9
Blackberry‐500	723.7 ± 266.7	249.1 ± 87.8	61.2 ± 38.4
Black mulberry‐250	1003.4 ± 252.7	302.1 ± 107.1	57.8 ± 33.1
Black mulberry‐500	1055.0 ± 447.3*	351.3 ± 83.0*	63.6 ± 29.5
Cranberry‐250	763.6 ± 168.1	286.4 ± 174.0	74.8 ± 47.5
Cranberry‐500	918.7 ± 202.0	364.2 ± 107.3*	67.8 ± 49.5

* *p* < .05 vs vehicle group.

**FIGURE 4:**
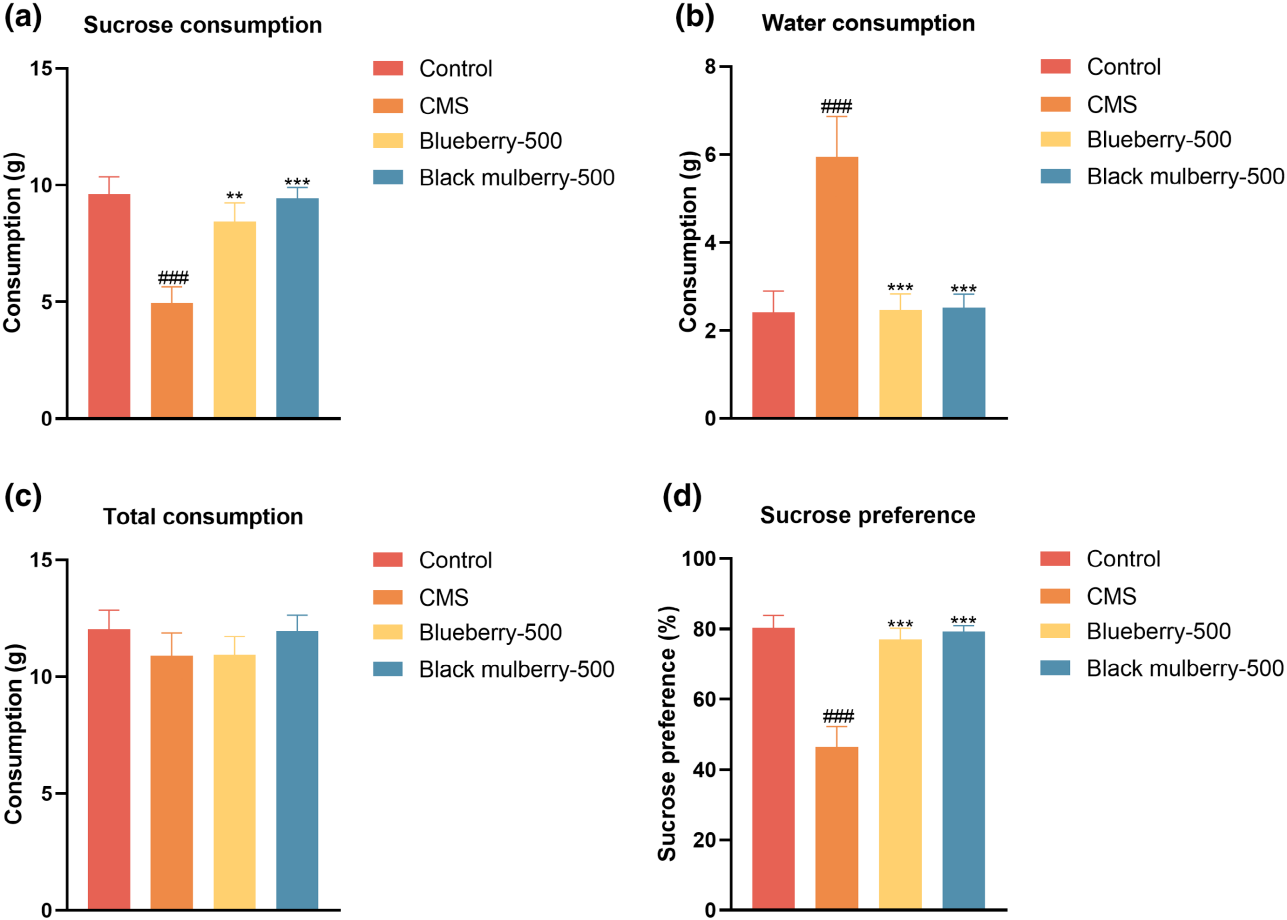
Effects of blueberry and black mulberry after chronic administration in the sucrose preference test. (a) Sucrose consumption. (b) Water consumption. (c) Total fluid consumption. (d) Sucrose preference (*n* = 8). Significance was assessed using a one‐way ANOVA followed by Dunnett's post hoc test. ^###^ *p* < .001 vs Control group. **p < .01 and ***p < .001 vs CMS group.

### 3.5 Effects of blueberry and black mulberry on depressive‐like symptoms in CMS mice

Subsequently, the CMS‐induced depressive‐like model has been performed to verify the antidepressant‐like effects of blueberry and black mulberry. As shown in Figure 4, CMS induced a reduction in sucrose consumption and sucrose preference, while blueberry and black mulberry increased the sucrose consumption and sucrose preference. In addition, CMS induced an increase of water consumption in mice, while blueberry and black mulberry decreased the water consumption. More importantly, blueberry and black mulberry did not exert any alterations in total fluid consumption. These data suggested that blueberry and black mulberry could reverse anhedonia, the core symptom of depression in mice.

**FIGURE 5:**
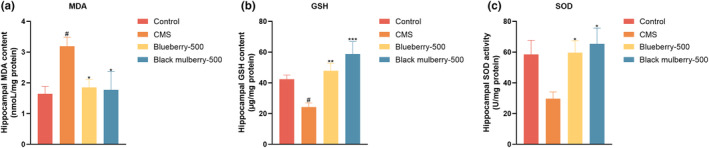
Effects of blueberry and black mulberry after chronic administration in CMS mice. (a) MDA, (b) GSH, and (c) SOD levels (*n* = 8). Significance was assessed using a one‐way ANOVA followed by Dunnett's post hoc test. ^#^ *p* < .05 vs Control group. *p < .05, **p < .01, and ***p < .001 vs CMS group.

### 3.6 Effects of blueberry and black mulberry on hippocampal MDA, GSH, and SOD levels

Oxidative stress damage was closely associated with the pathophysiology of depression. The levels of MDA were significantly increased in the hippocampus in CMS mice, while blueberry and black mulberry decreased the levels (Figure 5a). On the other hand, CMS decreased GSH content and SOD activity in the hippocampus, which was reversed by administration with blueberry and black mulberry (Figure 5b,c). These data indicated that blueberry and black mulberry improved the activity of oxidative stress in the hippocampus.

### 3.7 Effects of blueberry and black mulberry on hippocampal pro‐inflammatory cytokine levels

**FIGURE 6:**
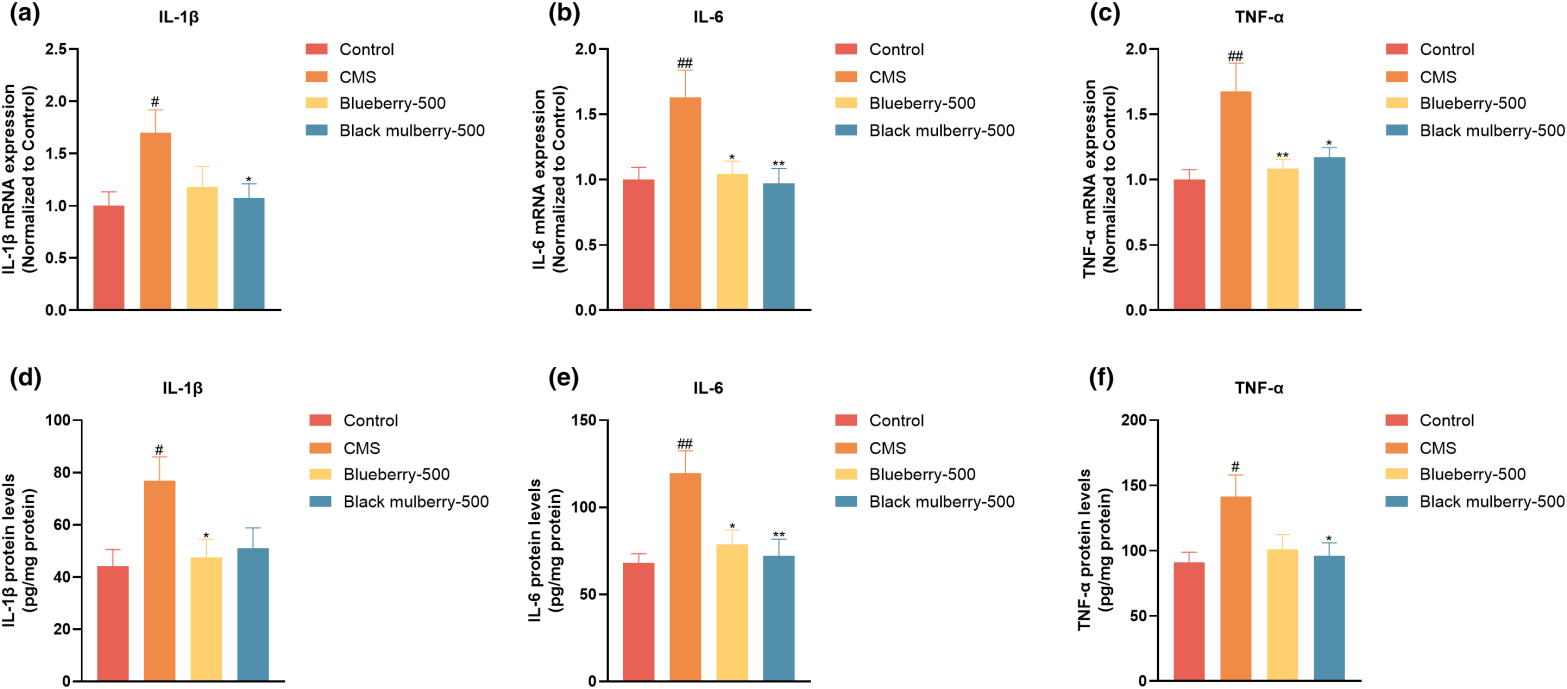
Effects of blueberry and black mulberry on hippocampal pro‐inflammatory cytokines in CMS mice (*n* = 8). (a) IL‐1β mRNA expression, (b) IL‐6 mRNA expression, and (c) TNF‐α mRNA expression. (d) IL‐1β protein levels, (e) IL‐6 protein levels, and (f) TNF‐α protein levels. (*n* = 8). Significance was assessed using a one‐way ANOVA followed by Dunnett's post hoc test. ^#^ *p* < .05 and ^##^ *p* < .01 vs Control group. *p < .05 and **p < .01 vs CMS group.

Depression is associated with activated inflammatory reactions in the central nervous system. Here, we measured the markers of inflammation following the administration of blueberry and black mulberry. The messenger RNA (mRNA) expression of pro‐inflammatory cytokines, including IL‐1β, IL‐6, and TNF‐α, was significantly increased in the hippocampal tissue in the CMS mice. On the contrary, black mulberry showed a significant reduction in IL‐1β, IL‐6, and TNF‐α expression, while blueberry showed a significant reduction in IL‐6 and TNF‐α expression (Figure 6a–c). On the other hand, the protein levels of IL‐1β, IL‐6, and TNF‐α were also significantly elevated in the hippocampal tissue after CMS procedure. In contrast, blueberry showed a significant reduction in IL‐1β and IL‐6 levels, while black mulberry showed a significant reduction in IL‐6 and TNF‐α levels (Figure 6d–f).

## 4 DISCUSSION

Previous research has extensively investigated the antioxidant and neuroprotective properties of various berries, including those studied herein. However, significant gaps remain, particularly in the breadth of the studies conducted. Predominantly, past research has concentrated on the effects of individual phenolic compounds or single extract within a single berry type, often neglecting comparative analyses across different berries. Additionally, apart from blueberries (Gapski et al., 2019; Oh et al., 2018; Spohr et al., 2022), scant attention has been paid to the potential of other berries to ameliorate depressive behaviors. Furthermore, inconsistencies in research outcomes are frequently noted due to variations in experimental methodologies, durations, animal models used, and observer differences (Fuchs & Fliugge, 2006). These disparities can lead to conflicting results concerning the antidepressant‐like effects of a given berry, and even more so when comparing across different berry types. To address these limitations, our study simultaneously compares the antioxidant capacities and antidepressant‐like effects of four distinct types of berries, aiming to provide a more comprehensive understanding of their therapeutic potentials.

Berry fruits are notably abundant in phenolic compounds, among which anthocyanins have been extensively investigated. The anthocyanin composition varies among berry species, offering valuable taxonomic insights. In this study, we focused on analyzing the anthocyanin profiles of selected berries. Anthocyanin‐rich extracts were obtained from these berries using ethanol extraction, followed by the identification and quantification of anthocyanin profiles using UPLC–Q Exactive/MS. The anthocyanins quantified in this study were selected based on the commercial availability of 15 standard anthocyanins, facilitating the creation of standard curves for accurate measurement. The findings of this study, while qualitatively consistent with those of prior research, showed quantitative differences that can be attributed to several factors. The variation in anthocyanin content is influenced not only by genetic and environmental conditions prior to and after harvest but also by the specific methodologies employed in the extraction and chromatographic analysis (Spinardi et al., 2019; Wang et al., 2020). For instance, we observed that blueberry and black mulberry contained high levels of cyanidin‐3‐galactoside (30,310.28 and 30,003.25 μg/g, respectively) and cyanidin (1061.13 and 3303.94 μg/g, respectively). Conversely, blackberry and cranberry demonstrated substantial concentrations of cyanidin‐3‐galactoside (17,598.43 and 3382.95 μg/g, respectively) and rutin (615.65 and 207.25 μg/g, respectively). These findings underscore the complex interplay of factors affecting anthocyanin profiles in berries and highlight the importance of standardized analytical approaches to facilitate comparative studies.

Depression is a mental disease associated with neurochemical, oxidative, and neuroinflammatory dysfunctions (Tian et al., 2022; Zhang et al., 2022). Despite considerable research into understanding the pathophysiology of depression, there are still no drugs that can completely reverse its progression (Karrouri et al., 2021). Natural products, including anthocyanin‐rich extracts from functional foods, show promise as dietary agents for the prevention and potential management of depression. Clinical and preclinical data suggest that the neuroprotective effects of berry fruits are associated with their polyphenolic compounds, particularly, anthocyanins (Maqsood et al., 2022; Traupe et al., 2018). For instance, anthocyanins have been reported to protect against oxidative stress‐mediated neuroinflammation (Banji et al., 2022). Cyanidin was efficacious in alleviating depression‐like symptoms, which were dependent on phosphoinositide 3‐kinase (PI3K)/protein kinase B (AKT)/forkhead box G1 (FoxG1)/fibroblast growth factor 2 (FGF‐2) signaling‐modulated neurogenesis enhancement (Shan et al., 2020). In this respect, we designed the current study to evaluate the potential antidepressant‐like effects of anthocyanin‐rich extracts from four common edible berries including blueberry, blackberry, black mulberry, and cranberry. The antidepressant‐like effects of anthocyanin‐rich extracts were evaluated in behavioral tests including forced swimming test, tail suspension test, and open‐field test. The investigation revealed that most berry extracts demonstrated antioxidative effects on serum according to the markers, such as MDA, GSH, and SOD. However, only blueberry and black mulberry exhibited significant antidepressant‐like activities in the forced swimming test and tail suspension test. This observation suggests that key anthocyanins, specifically cyanidin‐3‐galactoside and cyanidin, predominantly present in blueberry and black mulberry, may significantly contribute to their overall antidepressant‐like efficacy. Moreover, serum corticosterone levels, a commonly used biomarker for hypothalamic–pituitary–adrenal axis activity, were notably reduced by blueberry and black mulberry supplements. While blackberry at 250 mg/kg and cranberry at both 250 and 500 mg/kg did not influence corticosterone levels in serum. In neurotransmitter assay, black mulberry at 500 mg/kg increased 5‐HT and NE levels in the hippocampus, while blueberry at 500 mg/kg only elevated 5‐HT levels. Other berries did not exert any effect on monoamine neurotransmitters in the hippocampus. Despite both blackberry and cranberry containing cyanidin‐3‐galactoside, the content in blackberry was only 58% of that in blueberry, and in cranberry, it was merely 11% of content in blueberry. This differential content may explain the lack of observed antidepressant‐like effects in blackberry and cranberry, as previous research has identified cyanidin‐3‐galactoside as a crucial active component for cognitive and behavioral enhancement associated with blueberry consumption (Tan et al., 2014). Furthermore, the content of cyanidin in blackberry and cranberry was only 13% and 2%, respectively, of that in black mulberry. Cyanidin is known for its broad spectrum of biological functions, including antioxidant, anti‐inflammatory, and potential antidepressant‐like activities, in animal models (Shan et al., 2020). Therefore, our findings suggest a correlation between higher concentrations of cyanidin‐3‐galactoside and cyanidin and more potent antidepressant‐like effects in berries. It is important to note, however, that while cyanidin and related compounds likely play a significant role in the antidepressant‐like outcomes observed in the behavioral assays, other non‐cyanidin constituents, such as rutin, luteolin, and quercetin, may also contribute to these effects (Anjomshoa et al., 2020; Bappi et al., 2024; Mokhtari et al., 2023). Their potential additive, complementary, and synergistic interactions could enhance the therapeutic potential of these fruits for depressive disorders (Ma et al., 2018).

Given the superior performance of blueberry and black mulberry in the assays during acute supplement procedure, these berries were chosen for further biological evaluation in CMS‐induced depression models. The antidepressant‐like effects were initially assessed using the sucrose preference test, where a reduction in sucrose preference is recognized as a hallmark of anhedonia, a core symptom of depression. The results indicated that both blueberry and black mulberry significantly increased sucrose preference in mice, thereby attenuating anhedonia. Subsequently, we explored the potential neuroprotective effects of these berries in the hippocampus of CMS‐exposed mice. Both blueberry and black mulberry ameliorated CMS‐induced increases in MDA levels in the hippocampus, while concurrently reversing the CMS‐induced reductions in GSH and SOD levels. This observation suggests that these berries effectively attenuate oxidative stress associated with depression, which was consistent with the previous findings regarding blueberry and blackberry against brain oxidative stress (Krishna et al., 2019; Ma et al., 2024; Turgut et al., 2016). Beyond their antioxidant properties, the anti‐inflammatory effects of blueberry and black mulberry also likely play a role in their antidepressant‐like activities in CMS models. Notably, supplement with these berries reduced the production of pro‐inflammatory cytokines at both the gene and protein levels in the hippocampus, suggesting a capability to alleviate neuroinflammatory responses in depression. This is consistent with prior research indicating that blueberry can reduce oxidative stress and inflammation in BV2 microglial cells or in mice challenged with lipopolysaccharide (Lau et al., 2007). Additionally, a previous publication also indicated that extracts from black mulberry have been shown to protect against dopaminergic cell death by enhancing cellular antioxidant defenses and attenuating mitochondrial dysfunction (Tambe et al., 2023).

While our study demonstrates significant antidepressant‐like effects of blueberry and black mulberry, several limitations must be acknowledged. First, the scope of the present study was limited to specific anthocyanins, and other potentially active phenolic compounds in berries were not investigated. Second, the exact molecular mechanisms underlying the observed antidepressant‐like effects remain unclear, particularly how anthocyanins interact with neurochemical pathways. Third, the study relied on animal models, which may not fully replicate human depressive disorders, thus limiting the direct applicability of the findings to clinical settings. Additionally, the CMS model used in the study, while widely accepted, may not encompass the complexity of human depression. Future research should aim to elucidate the detailed molecular mechanisms of anthocyanins, including their interaction with neurotransmitter systems, inflammatory pathways, and oxidative stress markers. Clinical trials are also necessary to confirm the efficacy and safety of anthocyanin‐rich extracts from berries in human populations, and to explore their potential synergistic effects with other therapeutic compounds. Investigating the bioavailability and metabolism of anthocyanins in humans will further enhance our understanding of their therapeutic potential and inform the development of optimized dietary interventions for depression.

Finally, it should be noted that the preparation methods and dosages of berry extracts are critical factors that can significantly influence the reproducibility and consistency of experimental results. Variations in extraction techniques, solvent compositions, and extraction times can lead to differences in the concentration and composition of active compounds, such as anthocyanins. For instance, ethanol extraction, used in our study, might yield different anthocyanin profiles compared to other solvents like methanol or water. Furthermore, the ultrasonic‐assisted extraction method we employed can enhance the extraction efficiency but may not be directly comparable to those of conventional methods. Additionally, the dosages of berry extracts administered in animal studies can vary widely across different experiments, potentially affecting the outcomes. In our study, we used specific dosages based on preliminary experiments and existing literature; however, these dosages might not align with those used in other studies, leading to variability in the results. To address these issues, future research should aim to standardize extraction protocols and dosages to improve comparability.

## 5 CONCLUSIONS

Collectively, these findings suggest that blueberry and black mulberry exhibit significant antidepressant‐like effects, which are mediated by their anthocyanin components such as cyanidin‐3‐galactoside and cyanidin. These components contribute to the neurochemical regulation, antioxidative, and anti‐inflammatory actions of blueberry and black mulberry, underlying their therapeutic potential against depression. Berries rich in cyanidin‐3‐galactoside and cyanidin may be leveraged in the development of functional foods, dietary supplements, and pharmacological formulations for mental improvement. The integration of these berry extracts into functional foods and dietary supplements could provide a novel, natural approach to support mental health, particularly for individuals seeking alternative or complementary therapies to conventional antidepressant medications. Further researches are required to investigate the molecular mechanisms underlying the antidepressant‐like effects of anthocyanins, focusing on signaling pathways involved in neuroprotection, antioxidative stress, and anti‐inflammatory responses.

## AUTHOR CONTRIBUTIONS

**Jun Wang:** Formal analysis (lead); investigation (lead); writing – original draft (lead). **Jie Cheng:** Conceptualization (equal); investigation (equal); writing – original draft (equal); writing – review and editing (equal). **Ji‐Xiao Zhu:** Conceptualization (equal); formal analysis (equal); investigation (equal); writing – review and editing (equal). **Guang‐Hui Xu:** Conceptualization (equal); supervision (equal); writing – review and editing (equal). **Wei‐Feng Huang:** Conceptualization (equal); writing – review and editing (equal). **Li‐Tao Yi:** Conceptualization (equal); funding acquisition (equal); supervision (equal); writing – review and editing (equal).

## CONFLICT OF INTEREST STATEMENT

The authors declare that there is no conflict of interest.

## ETHICS STATEMENT

All experimental procedures received prior approval from the Huaqiao University Institutional Animal Care and Use Committee, Approval No. A2024002, and were conducted in strict accordance with guidelines set by the China Council on Animal Care.

## Data Availability

The data that support the findings of this study are available from the corresponding author upon reasonable request.
